# Genomic alterations on 8p21-p23 are the most frequent genetic events in stage I squamous cell carcinoma of the lung

**DOI:** 10.3892/etm.2014.2123

**Published:** 2014-12-09

**Authors:** JIUN KANG

**Affiliations:** Department of Biomedical Laboratory Science, Korea Nazarene University, Cheonan 330-718, Republic of Korea

**Keywords:** squamous cell carcinoma of the lung, copy number loss, homozygous deletion, tumor suppressor genes, microarray-comparative genomic hybridization

## Abstract

Genetic alterations in the early stages of cancer have a close correlation with tumor initiation and potentially activate downstream pathways implicated in tumor progression; however, the method of initiation in sporadic neoplasias is largely unknown. In this study, whole-genome microarray-comparative genomic hybridization was performed to identify the early genetic alterations that define the prognosis of patients with stage I squamous cell carcinoma (SCC) of the lung. The most striking finding was the high frequency of copy number losses and hemizygous deletions on chromosome 8p, which occurred in 94.7% (18/19) and 63.2% (12/19) of the cases, respectively, with a delineated minimal common region of 8p21.1-p23.3. More specifically, three loci of homozygous deletions at 8p23.1 were noted in 21.1% (4/19) of the cases. This region contains the following possible target genes, which have previously not been implicated to play a pathogenic role in stage I SCCs*: MSRA*, *MFHAS1*, *CLDN23*, *DEFB106A*, *DEFB105A*, *LOC441316*, *FAM90A7P* and *LOC441318*. These findings indicate that genetic alterations on chromosome 8p may be the first step in the initiation of genomic instability in early SCCs, and the newly identified genes in the 8p23.1 chromosomal region might be of interest for the study of the pathophysiology of stage I SCC, as potential targets for therapeutic measures.

## Introduction

Non-small cell lung cancer (NSCLC) has a high global cancer-related mortality rate (1). Two main types of NSCLC, namely adenocarcinoma (AC) and squamous cell carcinoma (SCC), are the most common subtypes. SCC of the lung is characterized by a complex pattern of cytogenetic and molecular genetic changes, and chromosomal aberrations are a hallmark of cancer cells, occurring at a high prevalence in SCC. Although a number of studies have been performed to evaluate genetic events associated with the development and progression of SCC (2,3), the molecular mechanism remains to be uncovered, and the identification of predictive markers is crucial.

Genetic alterations in the early stages of cancer have a close correlation with tumor initiation, and potentially activate downstream pathways that are implicated in tumor progression. With the recent advances of computed tomographic technology, the number of patients diagnosed with stage I lung SCC has been increasing (3). Although stage I SCCs are thought to be early-stage diseases and are treated primarily by surgery without adjuvant therapy, a considerable fraction of the patients with such SCCs have shown unfavorable outcomes following surgical treatment. Therefore, to improve the prognosis of those patients, it is necessary to identify suitable markers to select patients with poor prognosis, who would benefit from the use of adjuvant therapy following surgery (4). In particular, the identification of the genes that are altered during cancer initiation and progression can be valuable as therapeutic targets or prognostic indicators.

Studies have previously been performed to evaluate the genetic events associated with the initiation and progression of SCCs (5,6). However, little is known about the specific underlying genes that affect tumorigenesis in early stage SCCs. Furthermore, the genomic markers that predict aggressive clinical behavior of SCC remain to be identified. Therefore, in the present study, high-resolution array-comparative genomic hybridization (CGH) was conducted to identify early genetic alterations that define the prognosis of patients with stage I lung SCC.

## Materials and methods

### Tumor samples and DNA extraction

A total of 19 stage I SCCs from lung patients undergoing surgery as primary treatment, without previous radiation or chemotherapy, were analyzed. The demographic and pathological data, including age, gender and tumor stage, were obtained by a review of the medical records. All the patients were classified according to the World Health Organization classification histologic typing of lung carcinomas (7). This study has been reviewed and approved by the Institutional Review Board of the Chungnam National University Hospital (Daejeon, Korea); informed consent was obtained from the patients for the use of their tumors in the current study.

### Array-CGH experiment

The MacArray™ Karyo 4000 chips (Macrogen, Seoul, Korea) used in this study consisted of 4,046 human bacterial artificial chromosomes (BACs), which were applied in duplicate, and had a resolution of 1 Mbp (http://www.macrogen.co.kr) (8–11). Array-CGH was performed as described previously (12). Briefly, hybridizations were performed in a sealed chamber for 48 h at 37°C. Following the hybridization, array slides were scanned on a GenePix 4200A two-color fluorescent scanner (Molecular Devices Corporation, Sunnyvale, CA, USA) and quantification was performed using GenePix 4200A software (Molecular Devices Corporation). After scanning, the fluorescent intensities of the red and green channels were saved as two TIFF image files and the background was subtracted. Locally weighted scatterplot smoothing (LOWESS) normalization was applied to adjust for effects due to variation in the intensities between the red and green dyes. The breakpoint detection and status assignment of genomic regions was performed using a Gaussian model-based approach (GLAD) (13). A low-level copy number gain was defined as a log_2_ ratio >0.25, and a copy number loss was defined as a log_2_ ratio <−0.25. This threshold value was defined empirically as a value 3-fold that of the standard deviation calculated from hybridization experiments of 30 normal males to normal females.

### Statistical analysis for array-CGH

The Fisher exact test utilized two categories, normal and abnormal (loss and gain), with the null hypothesis that the relative proportions of the two categories would be the same in different groups. A multiple testing correction [Benjamini-Hochberg false discovery rate (FDR)] was applied to correct for the high number of false positive calls. R package version 2.2.1 of the Bioconductor Project (http://www.bioconductor.org) was used for detection of the frequency of gain or loss and statistical analysis.

## Results

### Whole genome array analysis of stage I lung *SCC cases*

**Figure 1:**
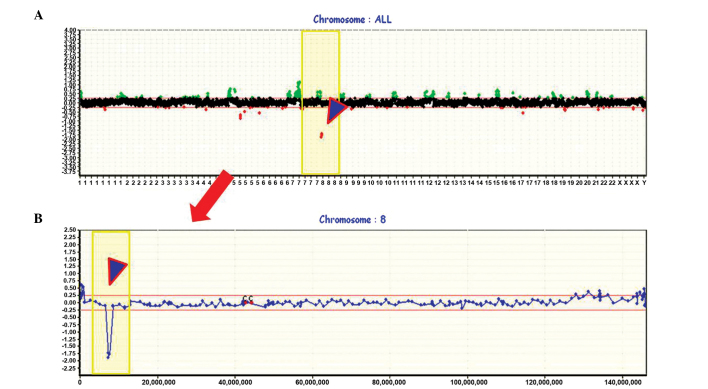
Examples of microarray-comparative genomic hybridization results from a patient sample (Tumor 2). (A) A log_2_ ratio >0.25 represents a genomic copy number gain, and a log_2_ ratio <−0.25 represents a genomic copy number loss. Clones are ordered from chromosome 1p to 22q. For the profiles, the x-axis represents the mapped position of the corresponding clone, and the intensity ratios are assigned to the y-axis. The homozygous deletions (HDs) at 8p23.1 are highlighted in yellow. (B) Genomic profiles of chromosome 8 from a patient sample (Tumor 2). The vertical lines indicate a ratio of <−1 in this bacterial artificial chromosome (BAC) clone, suggesting HD regions at 8p23.1. The HDs at 8p23.1 are highlighted in yellow.

A whole genome array-CGH was conducted to investigate DNA copy number alterations, and to identify new candidate genes in 19 patients with stage I SCC. Frequent gains were seen on chromosome arms 5p, 7p, 7q, 8q, 11q and 16p (>40% of patients), and frequent losses on 5q, 8p, 9q, 13q, 14q, 15q, 17p and 22q (>40% of patients; Fig. 1A). For the first step of the analysis, a decision was made to focus on chromosome 8p, the most frequently lost (94.7%, 18/19) and hemizygously deleted (63.2%, 12/19) region in stage I SCCs. More specifically, three loci of homozygous deletions (HDs) were displayed in 21.1% (4/19) of the cases. Due to the high frequency of chromosomal imbalances observed, it was hypothesized that deletions at 8p must be important and early genetic events in the pathogenesis of lung SCC, and that 8p may harbor tumor suppressor genes (TSGs) important for early SCCs. The minimal common region of chromosome 8p was identified to be located between BAC19_P21 and BAC181_E17 by genome-wide array-CGH.

### Chromosomal alterations of 8p21.1-p23.3 are the most common genetic alterations in stage I lung SCC cases

The most frequent regions of copy number alterations in these cases of SCC were defined more clearly, and narrowed down to 8p21.1-p23.3. A more detailed analysis of chromosome 8p identified three distinct regions of alterations across the chromosome. The minimal common region identified by array-CGH was located between BAC117_I18 and BAC250_C10. The delineation of 8p21.1-p23.3 chromosomal regions and possible target genes of the SCC cases are shown in Table I.

The first candidate locus spanned 82.0–93.2 kb in the 8p21.1-p21.3 region. According to the information archived in the human genome databases (http://genome.ucsc.edu/), it is flanked by the BAC clones BAC117_I18 and BAC176_L07, and contains 63 possible target genes (1.8 Mb segment). Notably, a high-frequency of copy number losses (−0.25>log_2_ ratio) and hemizygous deletions (−0.5<log_2_ ratio <−1) at these region were detected in 47.4% (9/19) and 10.5% (2/19) of the cases, respectively.

The second locus spanned 88.6–114.6 kb in the 8p22 region, and demonstrated a high frequency of copy number losses in 7 of 19 cases (36.8%). This locus comprised the putative TSGs of deleted in liver cancer 1 (*DLC1*), tumor suppressor candidate 3 (*TUSC3*) and microtubule-associated tumor suppressor 1 (*MTUS1*). Furthermore, the following possible target genes were detected that have not previously been considered to play a pathogenic role in SCCs: *NAT2*, *LOC392206*, *LOC137012*, *NAT1* and *PDGFRL*. A high frequency of hemizygous deletions (−0.5<log_2_ ratio <11) in this region was also noted in 26.3% (5/19) of the cases.

**Figure 2:**
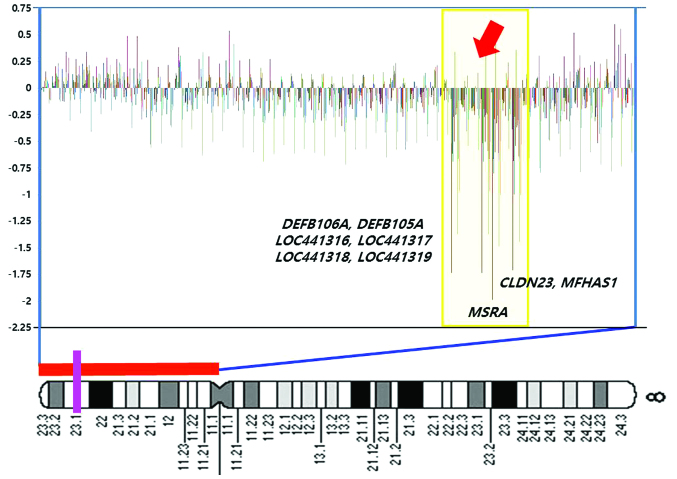
A diagram showing weighted frequencies (%) of squamous cell carcinoma cases on the short arm of chromosome 8. In the profiles, the y-axis represents the mapped position of the corresponding clone, and the intensity ratios are assigned to the x-axis. Cytobands are shown at the bottom of the ideogram. Vertical lines indicate the lowest locus of chromosome 8 in the bacterial artificial chromosome (BAC) clone containing the *MSRA*, *MFHAS1*, *CLDN23*, *DEFB106A*, *DEFB105A*, *LOC441316*, *LOC441317 (FAM90A7P)* and *LOC441318* genes. The homozygous deletions (HDs) at 8p23.1 are highlighted in yellow. Log_2_ ratio <−1 in this BAC clone, suggesting that homozygous deletions occurred at the *MSRA*, *MFHAS1*, *CLDN23*, *DEFB106A*, *DEFB105A*, *LOC441316*, *FAM90A7P* and *LOC441318* gene loci. Genes contained in clones are shown at the right.

The third locus was located distally in the 8p23.1-p23.3 region (87.5–104.7 kb). Notably, a high-frequency of copy number losses and hemizygous deletions at this region was detected in 89.5% (17/19) and 52.6% (10/19) of the SCCs, respectively. More specifically, three HD (−1< log_2_ ratio) loci at the 8p23.1 region were noted in 21.1% (4/19) of the cases. The first locus contained clones covering a region of ~112.8 kb, and comprised the *CLDN23* and *MFHAS1* genes, occurring in 15.8% (3/19) of the cases. The second locus spanning ~89.6 kb was found to contain the *MSRA* gene, placing it at the highest level of HD (21.1%, 4/19). The third locus spanned ~105.3 kb, which contains the *DEFB106A*, *DEFB105A*, *FAM90A18P*, *FAM90A9P*, *FAM90A10P* and *LOC441329* genes, also showing a high-frequency of HDs in the cases (21.1%, 4/19). The median span of the HDs was 7.5 Mb (range, 105.3–112.8 kb), and all HDs were located between BAC90_M06 and BAC234_K05. Representative genome profiles of HDs at the 8p23.1 region are presented in Fig. 1. Whole genome profiles are shown in the upper portion (Fig. 1A), and an individual profile of chromosome 8, including HDs at the 8p23.1 region, is presented in more detail below (Fig. 1B). An example of an individual profile showing HDs in the 8q23.1 region is presented in Fig. 2, and a schematic presentation of the cytogenetic bands, as well as map positions, is provided underneath.

## Discussion

In this study, whole-genome array-CGH showed that stage I lung SCCs display non-random patterns of co-occurring gains and losses. The most striking finding is characterized by a high frequency of copy number losses and HDs on the short arm of chromosome 8. Genomic changes on chromosome 8p have long been considered to be one of the major drivers of cancer progression, and are suspected to include critical TSGs in lung cancer (14–18). Previous investigations have focused on identifying somatic genetic mutations, including deletions and point mutations, of candidate genes on this region. Yan *et al* (5) reported that copy number deletions of chromosome 8p are one of the most prevalent genomic alterations in SCC of the lung, occurring at an incidence of 46%, and Sy *et al* (6) identified a preferential association of 8p loss with SCC pathogenesis. Furthermore, Shao *et al* (19) summarized the loss of heterozygosity (LOH) of 8p as an early hereditary event during the development of lung cancer. Allelic losses on 8p are also well described in other carcinomas, with most studies uncovering a complex pattern that cannot be reduced to a single minimally deleted region (20). In a study by Moore *et al* (21), array-CGH analysis revealed a high frequency of copy number losses at 8p (38%) in clear cell renal cell carcinoma, and the finding that the highest frequency of copy number alterations is on chromosome 8p has also described in prostate cancer (22). Notably, 8p allelic losses have also been detected in a relatively early stage during the pathogenesis of head and neck carcinomas (23). These results and the findings of the present study suggest that copy number losses on chromosome 8p are an important and early genetic event in the pathogenesis of lung SCC, and may harbor gatekeeper TSGs for these cancers (24).

On genomic analysis, chromosomal aberrations at the 8p21.1-p23.3 regions seem particularly noteworthy, due to the high-frequency of copy number losses and hemizygous deletions at this region, detected in 89.5 and 52.6% of the cases, respectively. Genetic alterations in the distal part of the 8p21.1-p23.3 region have been reported as early events frequently occurring in lung cancer. In addition, the size of these alterations, as well as their frequency, has also been reported to increase during lung cancer progression (25–27). These regions contain several interesting TSGs, the most attractive of which is the *DCL1* gene on 8p22. It is considered to be one of the prime target genes on 8p, and its reduced expression or HD has already been described in connection with various tumors, including lung cancer. Castro *et al* (28) reported *DLC1* as the most frequently methylated gene in lung tumors (50.0%) and described the methylation of this gene as an early event, associated with early differentiation and stage. Furthermore, a significant reduction or absence of DLC1 mRNA expression has been reported in 95% of primary NSCLC, and 58% of NSCLC cell lines (29). Emerging data from Kim *et al* on *DLC1,* which encodes a RhoA GTPase-activating protein, indicate that tumor suppressor loss in NSCLC is associated with genomic deletion or epigenetic silencing and loss of *DLC1* gene transcription (30). Additionally, Pils *et al* (31) summarized the epigenetic events in a frequently deleted region on chromosome 8p22 that influences the expression of *TUSC3*, a putative TSG in ovarian cancer. Similarly, Vaňhara *et al* (32) suggested that the expression of *TUSC3* epigenetically decreased in epithelial ovarian cancer, compared with that in benign controls, and provides prognostic information for patient survival. A recent study by Li *et al* (33) reported *MTUS1* as a potential tumor suppressor in gastric cancer. The results of these studies suggest that these gene changes may occur as early events in the development of several different types of cancer, and may also serve as a novel prognostic indicator for lung cancer.

Previous studies have noted that other tumor suppressors associated with early cancer development are likely to exist in the 8p21.1-p23.3 regions, in addition to the handful of identified candidates. In the present study, the following possible target genes were identified at 8p23.1 in a homozygous deleted region that was previously not considered to play a pathogenic role in SCC: *MSRA*, *MFHAS1*, *CLDN23*, *DEFB106A*, *DEFB105A*, *LOC441316*, *FAM90A7P* and *LOC441318*. Although involvement of these genes in the pathogenesis of SCC has not been previously mentioned, genetic mutations of these genes have consistently been reported in multiple tumor types (31–34). Lei *et al* (34) reported the *MSRA* gene as one of the well-annotated genes significantly downregulated in hepatocellular carcinoma (HCC), and suggested that it might play a role in the progression of HCC. Furthermore, Alonso Guervós *et al* (17) documented an association between the loss of the *MFHAS1* gene and lymph node metastases. Additionally, downregulation of the *CLDN23* gene in gastric cancer has also been reported (15,16). These findings support the theory that genetic mutations of these developmental genes may contribute to lung tumorigenesis at an early stage, and highlight the value of examining the genomes of pre-invasive stages of cancer at tiling resolution. Moreover, the newly identified target genes at the 8p23.1 HD chromosomal sites should provide important clues with regard to the genetic mechanisms underlying the initiation and progression of stage I SCC of the lung. Further investigation is required to validate and clarify the vital functions of these genes as novel targets for early SCCs, in larger studies using multiple samples.

In the present study, the previous findings concerning the 8p chromosome were significantly extended and the critical regions implicated in early SCC of the lung were firmly established. Furthermore, genomic analysis allowed the proposition of novel candidate genes that may be associated with the pathogenesis of stage I SCC cases. These findings suggest that genetic alterations on chromosome 8p are the first step in the initiation of genomic instability at the early onset of SCC and the newly identified target genes at the 8p23.1 HD chromosomal sites should provide important information with regard to the genetic mechanisms of the initiation and progression of stage I SCC.

## Figures and Tables

**Table I:** Chromosomal recurrent minimal regions of genetic alterations on chromosome 8p in 19 stage I SCCs.

Regions	Gene contained in clones	aLoss, n (%)	bHemizygous deletion, n (%)	cHomozygous deletion, n (%)
8p21.1-p21.3	*EXTL3*, *RC74*, *LOC340414*, *EXTL3*, *LOC389642*, *NKX3-1*, *NKX2-6*, *DPYSL2*, *DOCK5*, *GNRH1*, *KCTD9*, *CDCA2*, *TNFRSF10B*, *TNFRSF10C, RHOBTB2*, *CHMP7*, *LOC203069*, *LOXL2*, *FLJ10569*, *XPO7*, *NPM2*, *FGF17*, *EPB49*, *RAI16*, *FLJ22494*, *HR*, *DOK2*	8/22 (36.4)	2/22 (9.1)	3/22 (14.3)
8p22	*LOC392206*, *NAT2*, *DLC1*, *TUSC3*, *MTUS1, LOC137012*, *NAT1, PDGFRL*	8/22 (36.4)	5/22 (22.7)	1/22 (4.8)
8p23.1-p23.3	*MFHAS1*, *GATA4*, *NEIL2*, *LOC441338*, *FDFT1*, *CTSB*, *CLDN23*, *MFHAS1*, *MSRA*, *AGPAT5*, *DEFB106A*, *DEFB105A*, *LOC441316*, *FAM90A6P*, *FAM90A7P*, *LOC441318*, *CSMD1*, *LOC157693*, *LOC392169*, *FBXO25*, *INM01*, *FLJ00290*, *LOC401441*, *LOC389607*, *LOC157697*, *LOC442372*	8/22 (36.4)	2/22 (9.1)	3/22 (14.3)

a Alterations are defined by log_2_ ratio thresholds of −0.25 for copy number loss.

b Alterations are defined by log_2_ ratio thresholds of −0.5<log_2_ ratio <−1 for hemizygous deletion.

c Alterations are defined by log_2_ ratio thresholds of <−1 log_2_ ratio for homozygous deletion. Using this threshold, a frequency table was generated.

SCC, squamous cell carcinoma.
